# Robotic Surgery for Bladder Endometriosis: A Systematic Review and Approach

**DOI:** 10.3390/jcm12165416

**Published:** 2023-08-21

**Authors:** Marco Aurelio Pinho Oliveira, Thiers Soares Raymundo, Thiago Dantas Pereira, Ricardo José de Souza, Felipe Vaz Lima, Rudy Leon De Wilde, Leila Cristina Brollo

**Affiliations:** 1 Department of Gynecology, State University of Rio de Janeiro, Rio de Janeiro 20551-030, Brazil; thiersoares@gmail.com (T.S.R.); thiago_dantas@terra.com.br (T.D.P.); rsouzaj@gmail.com (R.J.d.S.); leilabrollo@gmail.com (L.C.B.); 2 Department of Gynecology, Cardoso Fontes Federal Hospital, Rio de Janeiro 22745-130, Brazil; 3 Department of Urology, Gaffrée e Guinle University Hospital, Rio de Janeiro 20270-004, Brazil; felipevaz.urologia@gmail.com; 4 Department of Gynecology, University Hospital for Gynecology, Pius Hospital, 26121 Oldenburg, Germany; de.wilde@yahoo.com

**Keywords:** bladder endometriosis, robotic surgery, systematic review, surgical treatment, minimally invasive approach

## Abstract

Introduction: Women with bladder endometriosis often present with more advanced stages of endometriosis. Robotic surgery has emerged as a promising approach to the management of bladder endometriosis. This systematic review aims to analyze the current literature on robotic surgery for bladder endometriosis and describe our systematic approach to surgical treatment. Methods: This review followed the PRISMA guidelines, which ensured a comprehensive and transparent approach to selecting and evaluating relevant studies. We conducted a thorough literature search to identify studies that investigated the use of robotic surgery for bladder endometriosis. Relevant databases were searched, and inclusion and exclusion criteria were applied to select eligible studies. Data extraction and analysis were performed to assess the outcomes and effectiveness of robotic surgery for the treatment of bladder endometriosis. Results: We did not find any randomized clinical trials with the use of robotics in the treatment of bladder endometriosis. We found only two retrospective studies comparing robotic surgery with laparoscopy, and another retrospective study comparing robotic surgery, laparoscopy, and laparotomy in the treatment of bladder endometriosis. All the other 12 studies were solely case reports. Despite the lack of robust evidence in the literature, the studies demonstrated that robotic surgery is feasible and is associated with reduced postoperative pain, shorter hospital stays, and faster recovery. Conclusions: The utilization of robotic technology is a promising option for the surgical management of bladder endometriosis. We advocate a surgical systematic approach for the robotic treatment of bladder endometriosis. Robotic technology, with its 3D vision, instrumental degrees of freedom, and precision, particularly in suturing, may provide potential benefits over traditional laparoscopy.

## 1. Introduction

Deep endometriosis (DE) is the presence of endometrial-like tissue outside the uterine cavity, infiltrating the peritoneum deeper than 5 mm [1]. Frequently, DE involves the uterosacral ligaments (66%), the vagina (17%), the intestine (9.5%), and the bladder (7.5%) [2]. When the urinary tract is compromised, the bladder and ureter are affected by 70 to 85% and 9 to 23%, respectively [3]. Bladder endometriosis (BE) is defined by the implantation of endometrial tissue into the detrusor muscle, affecting mostly the base and dome. In some cases, it can affect the ureteral ostium.

Women with BE usually present with more advanced stages of endometriosis, including in extragenital sites, and 35% have urinary symptoms, such as urinary frequency, pain, and/or bleeding [4]. Some women may also experience urinary urgency, but less frequently. Women with dyspareunia, dysmenorrhea, and pelvic pain associated with those urinary complaints provide strong evidence of BE.

The vaginal exam may identify palpable nodules or thickness at the anterior vaginal wall in some patients with BE [5]. Nevertheless, ultrasound (US) is a valuable tool in BE diagnosis because it is widely available, has low cost, has no radiation exposure, and has 62% sensibility and 100% specificity in detecting BE. Magnetic resonance imaging (MRI) is excellent in mapping pelvic endometriosis with high accuracy in detecting BE, with 64% and 98% of sensibility and specificity, respectively [3].

Hormonal therapies may be used as a first step to control BE symptoms, but patients usually do not respond adequately due to the detrusor’s desmoplastic reactions [6]. Therefore, when medical therapies fail, a surgical approach with complete removal of the bladder lesion should be performed to alleviate the symptoms and decrease the risk of recurrence [7]. Two surgical procedures are described in the literature: transurethral resection (TUR), partial cystectomy, and sometimes a combination of both [6]. Nevertheless, TUR alone should not be used because endometriosis grows from outside (serosa and detrusor) toward the bladder mucosa, which makes it unachievable to completely excise the lesion [8,9]. Therefore, partial cystectomy seems to be the best approach in women with BE. Minimally invasive surgery (laparoscopic or robotic-assisted) is the preferred approach because of several benefits. It is associated with a lower incidence of surgical morbidity, less postoperative discomfort, and a shorter length of hospital stay.

Due to the innovative features of robotic technology, which include an expanded three-dimensional view, a more comfortable working position for the surgeon, and more flexible and precise movements, it is possible to overcome the inherent limitations of conventional laparoscopy. As a result, it is appropriate for more complicated procedures, where extensive dissection and adequate restoration of the anatomy are required, such as endometriosis [10,11]. Some cases of urologic lesions, intestinal involvement, and also widespread peritoneal implants may benefit from a robotic approach [12].

### Anatomical Considerations and Endometriosis Bladder Presentations

The anatomical study of the bladder and its relationships is important to the knowledge of the main surgical steps of the treatment of bladder endometriosis (BE). It allows the surgeon to avoid undesired injuries and provide a better outcome with increased preservation of the bladder and its functionality.

In adult females, the bladder is a pelvic organ overlaid partially by the peritoneum on the superior surface and is reflected over the uterus to form the vesicouterine reflection, a very common site for BE. Incision of the peritoneum at this level allows access to the posterior bladder wall and its dissection from the vagina, which is firmly attached to the base of the bladder. Anteriorly and laterally, the bladder is surrounded by fat up to the pubis and a virtual space, and this virtual space (Retzius or retropubic space) can be created by the dissection of the transversalis fascia anteriorly, providing access to the anterior wall of the bladder [13].

The anterior parietal peritoneum, lateral to the bladder, forms the superior border of the lateral paravesical space that creates the broad ligament in females. When it is dissected medially, it is possible to access the paravesical space at each side down to the iliac vessels, obturator fossa, and levator ani muscles. This maneuver is useful in the complete resection of a lesion that affects the bladder lateral wall, and it allows a tension-free suture [14].

The bladder has an ovoid shape and its tetrahedral form when fully filled holds around 500 mL. The three layers of smooth muscles (inner longitudinal, middle circular, and outer longitudinal) are considered the main structure of the bladder wall, and they are best known as the detrusor muscle. There is an anchor to the anterior abdominal wall, called the urachus, made of longitudinal smooth muscle bundles that are localized in the bladder apex [15,16].

We can divide the bladder into four anatomical areas: apex, base, body, and inferolateral surface. Both ureters enter the bladder wall posteriorly in a diagonal direction and form the ureteral orifice after penetrating the detrusor for 1.5 to 2.0 cm. The trigone is localized between the two ureteral orifices and is composed of longitudinal smooth muscle fibers and the urethra orifice. All these structures are at the base of the bladder.

The lymphatic drainage passes to the external iliac, and some anterior and lateral drainage may go through the obturator and internal iliac nodes, whereas portions of the bladder base and trigone may drain [17].

Bladder innervation is made basically by parasympathetic stimulation, which leads to the contraction of the detrusor muscle and sympathetic stimulation, which leads to relaxation and facilitates expansion. It comes from the inferior aortic, hypogastric, and pelvic (bladder) plexus [18].

Bladder blood supplies come from the internal iliac vessels as the superior and inferior vesical arteries. The first is a branch from the obliterated umbilical artery. The inferior vesical artery is usually a branch from the uterine or vaginal arteries which also arise from the internal iliac vessels [14].

## 2. Methods

A literature review was conducted following the “preferred reporting items for systematic reviews and meta-analyses” (PRISMA) checklist [19] to identify articles using the PubMed, EMBASE, Lilacs, Cochrane Library, SCOPUS, and Web of Science databases with the terms “robotic surgery” OR “robotic” AND “bladder endometriosis”, with no language or date restrictions. Additionally, a search for gray literature was conducted in the OpenGrey database (http://www.opengrey.eu/search, accessed on 19 March 2023), Google Scholar (accessed on 19 March 2023), and WorldCat (accessed on 19 March 2023).

The inclusion criteria were: (1) full-text articles, (2) case reports or case series, and (3) interventional studies that analyzed the technique, postoperative follow-up, and surgical complications of robotic bladder endometriosis resection. Cases with ureteral involvement were not included. Reviews and duplicate studies were also excluded.

The search was independently performed by two authors who extracted pertinent data from eligible articles, which included information such as author, year of publication, research objective, study design, sample size, assessment and analytical methods, and results. In cases of disagreement, the data were evaluated by a third author.

A total of 49 articles (PubMed) were initially identified. After the duplicates were removed, the titles and abstracts checked, and the full text reviewed, 25 studies met the predetermined inclusion and exclusion criteria. Out of the initial 25 articles, 11 were excluded as they did not report bladder endometriosis, robotic surgery, or mention any follow-up information. As a result, 12 articles were included for further analysis. A secondary search was conducted in the bibliographical references of the selected articles to identify other sources not detected through the initial search. Three articles were added, making a total of fifteen.

**Figure 1:**
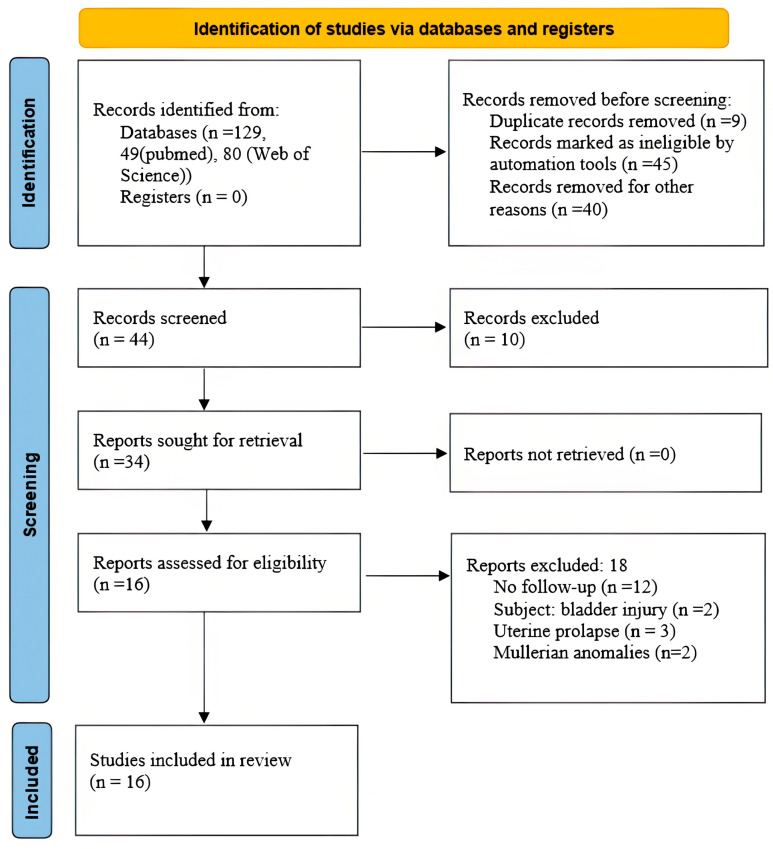
Flow diagram of literature search.

In the Web of Science database, a total of 80 articles were initially identified. After removing duplicates, reviewing titles and abstracts, and conducting a full-text review, 19 studies were found to meet the predetermined inclusion and exclusion criteria or were duplicated with the PubMed search. Of these, 18 were excluded, as they did not report bladder endometriosis, robotic surgery, or provide any follow-up information. Consequently, only 1 article met the criteria and was retained for further analysis (Figure 1).

## 3. Results

Endometriosis surgery must balance preserving function and fertility while achieving complete excision and preventing complications during and after surgery. A complex decision such as this one demands surgical judgment grounded in knowledge of the anatomy and pathology of endometriosis [20].

Minimally invasive approaches such as laparoscopic and robotic surgeries are preferred over laparotomy. Not many studies compared conventional laparoscopy (CL) with robotic surgery in the treatment of endometriosis. Saget et al., assessing perioperative results of robotic surgery (RS) in the context of DE, observed no increases in blood loss or in peri- or postoperative complications [21]. Sotto et al., in a randomized clinical trial (RCT) with 73 women, observed no differences in operative time, blood loss, conversion to laparotomy, perioperative complications, and quality of life [22]. Nezhat and Sirota, in a retrospective study, observed after body mass index stratification that obese patients had significantly longer surgical time in RS compared to CL: 282.5 min [range, 224–342 min] for RS versus 174 min [range, 130–270 min] for conventional laparoscopy (*p* < 0.05) [23].

Studies of RS and bladder endometriosis are presented in the literature, mostly in the form of case reports. We found twelve case reports—one of them is a video article (Table 1) and three are retrospective studies (Table 2).

All case reports showed that surgical management was feasible and had good results using robotic surgery [24,25,26,27,28,29,30,31,32,33,34,35,36]. Retrospective studies evaluated heterogeneous outcomes but, in general, concluded that RS was feasible and safe. When comparing blood loss, surgical time, conversion to laparotomy, and incidence of complications, there were no differences between RS and CL [36,37,38,39].

**Table 1:** Case Reports.

Author, Year	Objective	Conclusion
Senner, 2006 [34]	To describe a simultaneous transurethral and laparoscopic partial cystectomy and robot-assisted bladder reconstruction.	Patient remained symptom-free at last follow-up. No intra- and postoperative complications.
Liu, 2008 [30]	To reveal a surgical approach for the treatment of a patient with severe pelvic and infiltrative bladder endometriosis with mucosal involvement using robotic-assisted laparoscopic excision and cystotomy repair	Approximately 100 mL blood loss. No intra- and postoperative complications.
Chammas, 2008 [29]	To evaluate the feasibility and safety of robot-assisted laparoscopic partial cystectomy for the treatment of rectal and bladder endometriosis.	Robotic-assisted partial cystectomy with concomitant excision of endometrial nodules from the rectum and ovarian cyst is feasible and safe.
Bot-Robin, 2011 [31]	To evaluate the feasibility of robotic-assisted laparoscopy for deep pelvic endometriosis nodule resection.	Six patients were included. No conversion to laparoscopy or laparotomy. No intraoperative complication. One patient with vesicovaginal hematoma and bilateral pyelonephritis, on the 14th postoperative day. Average time 173 min, blood loss < 100 mL.
Siesto, 2014 [36]	To evaluate the feasibility of robotic surgery for the management of deep endometriosis.	Five bladder resections were performed. Neither intraoperative complications nor conversion to laparotomy occurred.
Abo, 2017 [27]	To assess the feasibility of deep endometriosis surgery using robotic assistance, benefits, and limits of this approach.	Surgical management is feasible using robotic assistance.
Moawad, 2018 [35]	A technical video showing a step-by-step approach for the resection of considerable involvement of the bowel and bladder with deeply infiltrative endometriosis using robotic platform	No intra- and postoperative complications.
Tan, 2018 [33]	To report an infertility case of deep-infiltrating bladder endometriosis following robot-assisted surgery and modified gonadotropin-releasing hormone agonist (GnRHa) treatment.	Blood loss 100 mL. No intra- and postoperative complications. Robot-assisted complete resection of deep endometriosis and bladder repair immediately followed by GnRHa therapy and medical assistance improves reproductive outcomes efficiently in women with endometriosis-associated infertility
Tamhane, 2020 [26]	To describe the surgical approach for deep endometriosis of the ureterovesical junction and bladder reconstruction.	In patients with DIE of the bladder, bimodal visualization might be needed to delineate the extent of the disease.
Wei, 2021 [28]	To report a transvesical dissection of a 2 cm bladder mass in the bladder wall approximately 2 cm from the right ureteral orifice, with the aid of intraoperative cystoscopy	The procedure was completed successfully with no need for open conversion. The total duration of the operation was 1.8 h and intraoperative blood loss was low.
Badahur, 2021 [25]	To describe the dual approach for bladder endometriosis	Robotic approach seems safer and easier in this complex surgery owing to dense adhesions in such cases.
Guan, 2022 [24]	To demonstrate tips and tricks for the successful execution of robotic-assisted resection of a large bladder trigone endometriosis nodule.	Robotic-assisted resection of bladder trigone endometriosis with cystoscopic guidance provides optimal bladder trigone and ureteral preservation.

**Table 2:** Retrospective Studies.

Author, Year	Objective	Methods	Conclusion
Le Carpentier, 2016 [38]	To compare robotic and laparoscopic surgery for bladder endometriosis.	There were 15 patients in the robotic surgery group and 22 in the conventional laparoscopy group. The median age was 29 years ± 7 years. The symptoms were similar in the two groups.	Robotic surgery in the surgical treatment of bladder endometriosis as compared to laparoscopy does not seem to have an adverse effect on either the risk of recurrence or the occurrence of intra- and postoperative complications.
Di Maida, 2020 [37]	To present the surgical techniques and the postoperative outcomes in women treated with robotic excision for deep endometriosis involving the urinary tract.	There were 74 consecutive patients enrolled. Of these, 28 (37.8%) patients underwent conventional laparoscopy and 46 (62.2%) robotic surgery.	Concomitant involvement of bowel and genital systems was registered in 14 (30.4%) and 32 (69.5%) patients, respectively. No conversions to laparotomy were recorded. Overall, 5 (10.9%) patients experienced postoperative complications, of which only 1 was a major complication.
Philip, 2021 [39]	To describe the surgical management and risks of postoperative complications of robotic surgery in urinary tract endometriosis.	A total of 172 patients underwent surgery by conventional laparoscopy (74.1%), 34 by robotics (14.7%), and 26 by laparotomy (11.2%).	The surgical management of bladder endometriosis is usually feasible and safe.

Therefore, the studies demonstrate the feasibility of robotic surgery for deep endometriosis involving the bladder and have shown comparable results with conventional laparoscopy for the treatment of endometriosis.

The additional costs account for not only the expense of the equipment itself but also its maintenance, the requirement for specially trained workers, and the lengthier time spent in the operating room. However, if robotic technology leads to a higher proportion of cases being performed by minimally invasive techniques, with the potential results of decreased postoperative morbidity and fewer recovery days off work, then this cost may be outweighed by the benefit to the general public [40].

### Systematic Approach for Bladder Endometriosis Resection in Robotic Surgery

The preparation of the patient with bladder endometriosis for robotic surgery should follow the same principles as for the surgical treatment of DE in other sites. The patient should be positioned with open legs to facilitate access to the perineum. Trendelenburg should be used only to the point that the small intestines do not fall into the pelvis. We usually start with maximum cephalodeclive and pull the small bowel out of the pelvis. In the sequence, we ask an assistant to decrease the Trendelenburg level until we find the best position, with the least cephalodeclive as possible.

**Figure 2:**
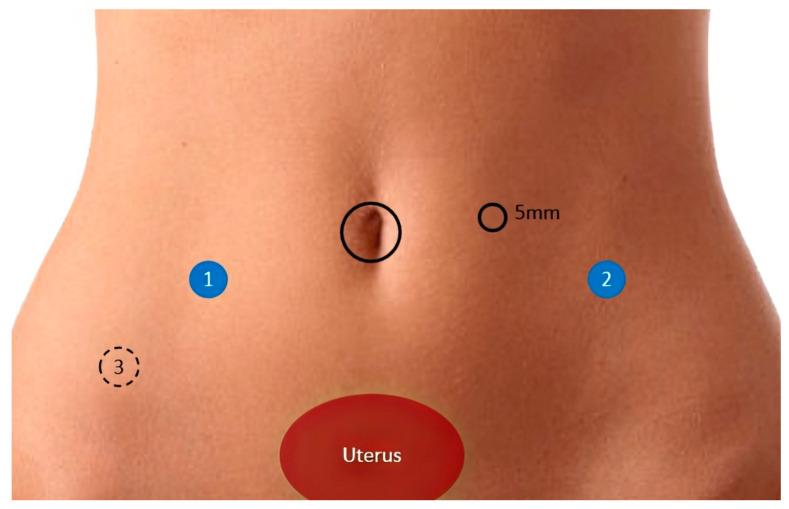
Placement of the portals (1 on the right and 2 on the left). Most cases are performed with only two robotic arms, in addition to the optics arm (placed at the umbilicus). We rarely need the placement of a fourth robotic arm (number 3). We use a 5 mm laparoscopic portal, which is used by the assistant.

The next step is to decide where to make the port placements. Another important change in our practice, especially after we started using the Xi DaVinci platform (Intuitive Surgical^®^, Sunnyvale, CA, USA), is to almost never use the fourth robotic arm (Figure 2). By making one fewer incision, we can reduce the cost, improve aesthetics, and maybe reduce the frequency of port complications, such as hernias. The camera arm is always inserted through the belly button in all cases of endometriosis, including BE.

The instruments used for the robotic procedure are monopolar scissors, the fenestrated bipolar, and a needle-holder. For cost reduction, we avoid using two needle holders for suturing and we use, instead, the bipolar itself as a suture assistant (plus one needle holder).

Once the camera is docked, we inspect the abdominal and pelvic cavities. With a good preoperative image assessment, usually we have a good correlation with the surgical findings, and we can follow the presurgical plan. However, it is useful to perform a cystoscopic evaluation for the exchange of information between the urologist and the gynecological surgeon when working with a multidisciplinary team.

**Figure 3:**
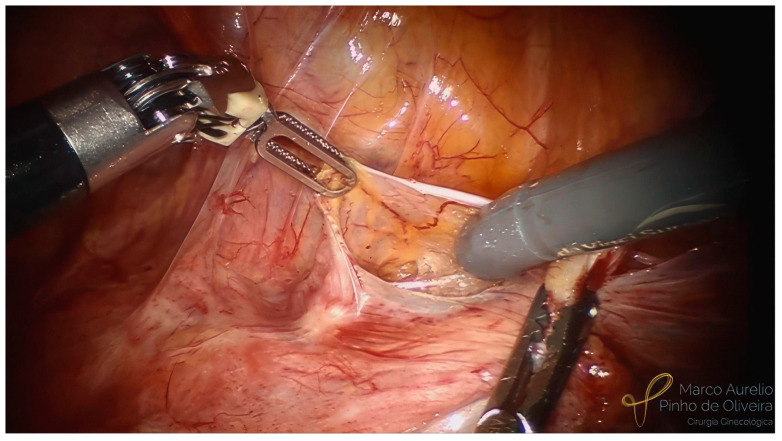
Dissection of the right paravesical space.

**Figure 4:**
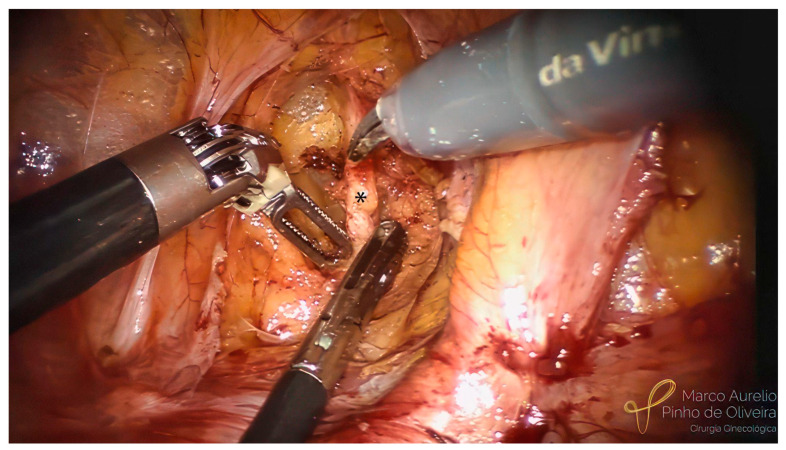
Dissection of the right paravesical space. Identification of the right umbilical ligament (*).

**Figure 5:**
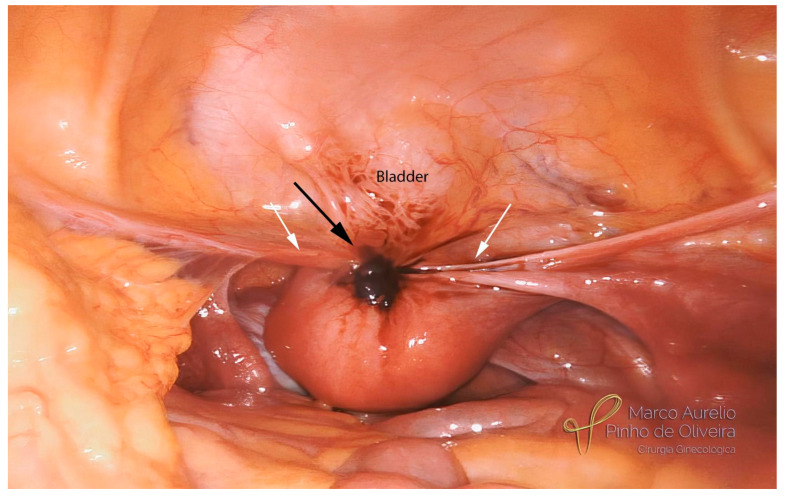
Bladder endometriosis (black arrow) causing retraction of the round ligaments (white arrows) and myometrial infiltration.

**Figure 6:**
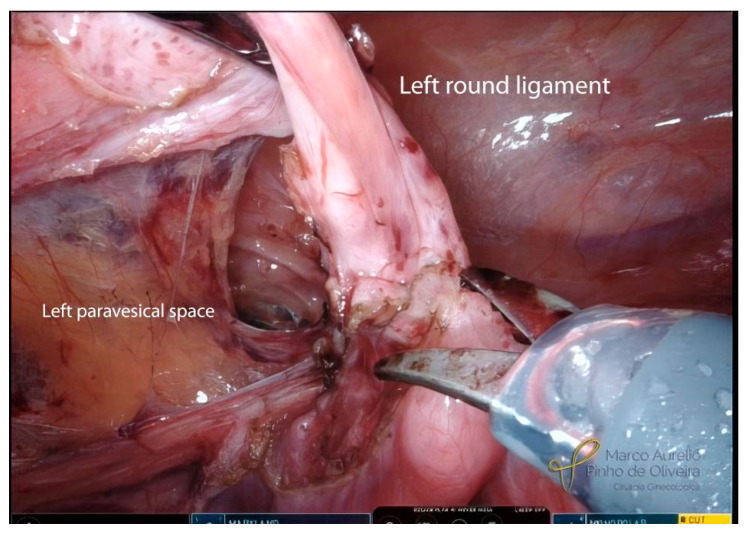
Resection of the left round ligament infiltrated by endometriosis.

The systematic approach for the resection of bladder endometriosis, as well as for most endometriotic lesions, should be performed from the healthy tissue to the diseased area. After the identification of the BE, we start developing the paravesical space on the right side with an opening of the anterior aspect of the broad ligament (Figure 3). We always try to identify the umbilical ligament (Figure 4) before dissecting caudally in the direction of the pelvic floor. The association between BE and endometriosis (with retraction) of the round ligament is high (Figure 5) [41]. If it is affected, we remove the area of the round ligament infiltrated by the disease (Figure 6). The same steps are performed on the left side.

**Figure 7:**
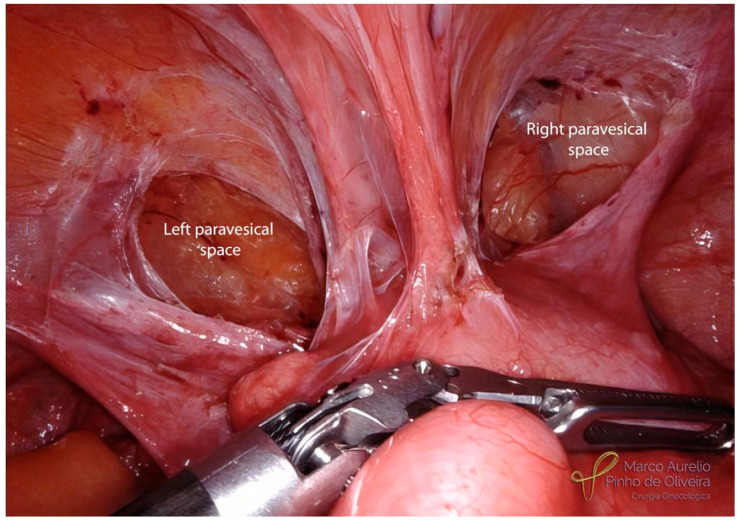
Both paravesical spacesare dissected and the lesion is “centralized”. No need to remove the round ligaments in this case.

When feasible, we try to identify the normal vesicovaginal space caudal to the endometriotic lesion, making a retrograde (caudal–cranial) dissection of this space. We routinely use a uterine manipulator with a vaginal delineator, as it is very helpful to identify the vaginal wall, making the dissection of the vesicovaginal space much easier. These dissections are made on both paravesical spaces (right and left), with the objective of “centralizing” the disease (Figure 7), a concept already described by our group for the resection of endometriosis of the posterior compartment [42]. It is important to note that “paravesical space” does not exist in the anatomical nomenclature, which may lead to discrepancies among surgeons. However, some authors consider the lateral area to the umbilical arteries as the paravesical space (paravesical fossae), and the more medial part as the true retropubic space [43].

**Figure 8:**
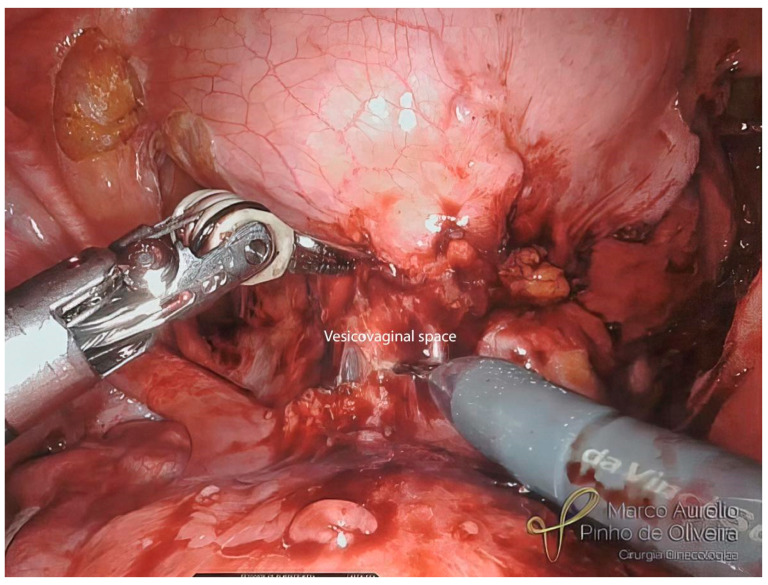
Dissection of the vesicovaginal space with the bladder partially filled.

After both paravesical spaces have been dissected and the lesion centralized, we begin the dissection of the vesicouterine space, with the aim of mobilizing the lesion from the uterus and vagina and leaving it only stuck to the bladder. This dissection can be obtained with small cuts with monopolar scissors in high-power pure cut mode (level 5/6 in the Xi console) in the cranial–caudal direction. During this step, we can fill the bladder with 100 to 150 mL of saline to better delineate the bladder dome and facilitate the dissection of the vesicovaginal space (Figure 8).

**Figure 9:**
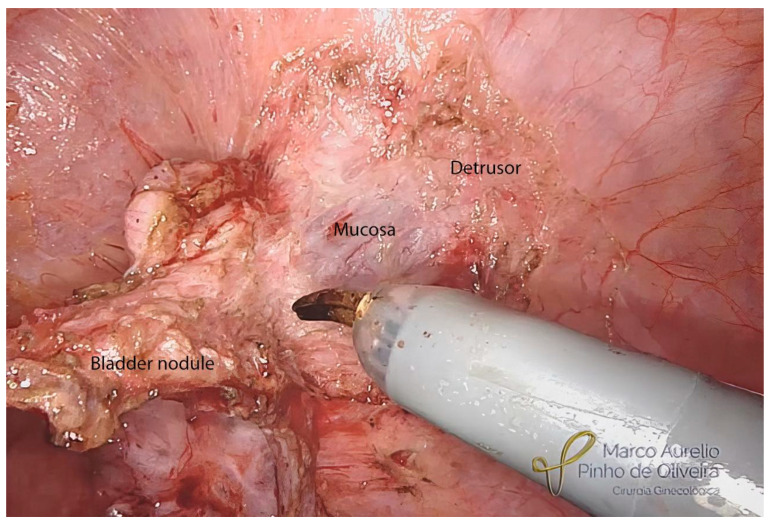
Shaving of the bladder endometriosis nodule.

**Figure 10:**
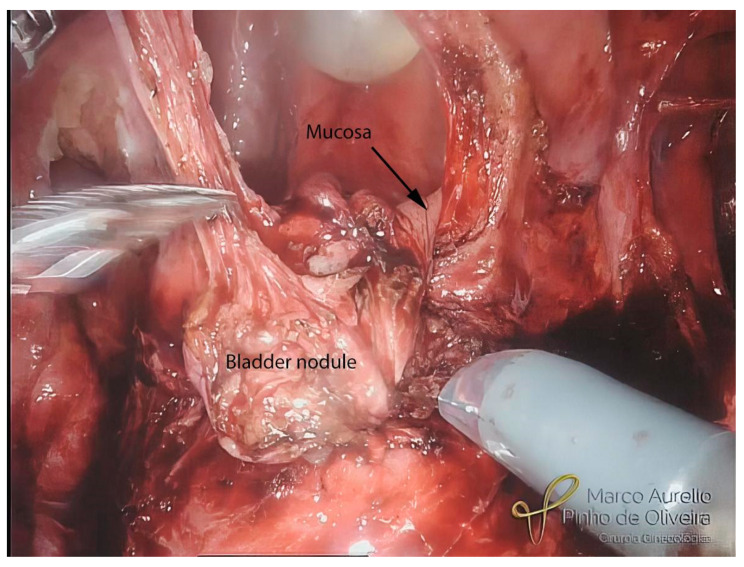
Opening of the bladder and resection of the nodule up to normal mucosa.

**Figure 11:**
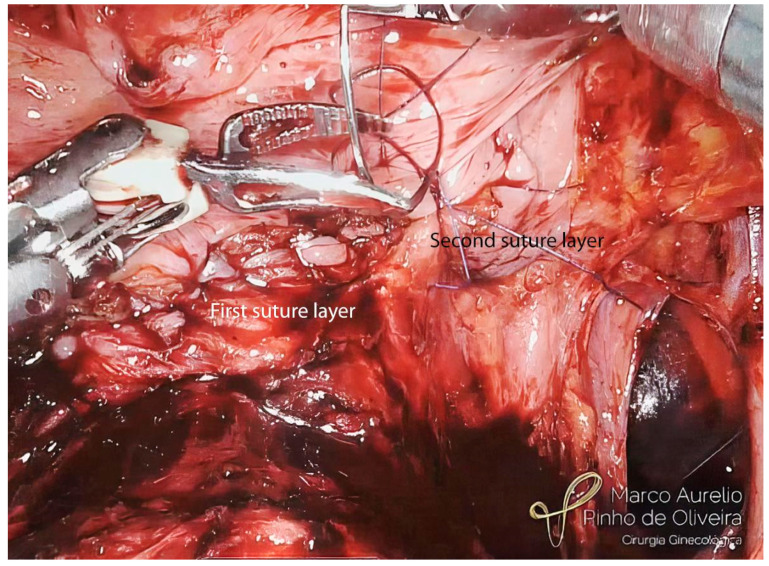
Opening of the bladder and resection of the nodule up to normal mucosa.

With the endometriotic lesion already mobilized, we make a shallow incision with monopolar scissors around the nodule to delimitate the area to be removed. Whenever possible, we try to shave the nodule, avoiding opening the bladder mucosa, mostly when intraoperative cystoscopy is negative for mucosal infiltration (Figure 9). When the mucosa is affected, we open the bladder and finalize the nodule resection, evaluate the mucosa (Figure 10), and always check the ureteral ostia. Except for lesions infiltrating the trigone, we do not routinely insert a double “J” catheter in the surgical treatment of BE. We close the bladder in two planes. For the mucosa, we use polyglycolide 4.0 in a running fashion and for the muscle layer, we use 4.0 polyglactin (or polydioxanone 4.0), also in a running fashion (Figure 11).

## 4. Final Conclusions

The bladder is the urinary tract area most commonly affected by endometriosis [3]. Since BE pain overlaps with endometriosis pain symptoms from other locations and other pathologies such as bladder pain syndrome, a thorough evaluation is necessary. Seeking consultation with a urologist, pelvic physiotherapist, and/or pain specialist can assist in evaluating the differential diagnosis. It is crucial to understand and address patient expectations, regardless of whether they are experiencing pain, infertility, or an advanced disease with no complaints.

Mapping disease extension is of utmost importance for either medical or surgical management. High-quality imaging exams, such as pelvic MRI or ultrasound, are important tools for diagnosis. Consultation with a urologist is advised for surgical planning. Identifying BE nodules preoperatively is important for patient counseling and for identifying cases where full-thickness excision is indicated and its distance to the ureteral ostia. When the disease is located either too close to one ostium or in the vesical trigone, the surgical team should be prepared for the necessity of ureteral stents or even reimplantation.

Urinary tract endometriosis coexists with deep infiltrative endometriosis in other sites. When surgery is indicated, a multidisciplinary approach is recommended. A minimally invasive approach is preferred over laparotomy, as it provides adequate visualization of both disease lesions and anatomical structures, besides having all the known advantages of laparoscopic or robotic surgery. Transurethral resection must be avoided, as it does not provide complete nodule excision, favoring the maintenance of pain and disease persistence.

Specifically, regarding urinary tract endometriosis, the suturing capability and instrumental degrees of freedom in robotic surgery provide possible advantages over conventional laparoscopy. Robotic suturing dexterity can overcome difficult angles the surgeon might find during bladder repair, especially when trigone lesions are resected. When ureteral reimplantation is necessary, robotic surgery, with its 3D vision and endowrist motion, may play an important role. With robotic aid, in cases where the distal portion of the ureter is affected and ureterolysis is not feasible, a good end-to-end anastomosis is achievable, even if it is a few centimeters away from entering the bladder. These cases are usually primarily managed by ureteroneocystostomy.

Abdominal drainage is usually performed when the resection area is extensive, or the trigone suture was considered difficult. Bladder suturing can be performed in one or two layers, according to the surgeon’s preference. We usually perform a two-layer suture and perform a bladder distention test, filling it with saline and methylene blue dye to ensure that there is no leakage. Extensive bladder mobilization might be necessary to ensure that the suture is tension-free, to decrease the risk of dehiscence. Foley catheterization in the postoperative period depends on the surgeon’s decision and pelvic nerve trauma during surgery. It should be considered, as bladder atonia will lead to overdistention and may put the sutures at an increased risk of dehiscence. Antibiotics are not regularly used. We usually leave the catheter for a period of 5 to 15 days, depending on the extent of the surgery.

A robotic platform is an important tool in endometriosis surgery. Robotic training is of paramount importance for increasing robotic usage in gynecology. With the development of newer platforms, it is likely the number of surgeries will increase as the number of certified surgeons increases.

## Data Availability

No new data were created.

## References

[1] Koninckx P.R., Martin D.C. (1992). Deep Endometriosis: A Consequence of Infiltration or Retraction or Possibly Adenomyosis Externa?. Fertil. Steril..

[2] Chapron C., Fauconnier A., Vieira M., Barakat H., Dousset B., Pansini V., Vacher-Lavenu M.C., Dubuisson J.B. (2003). Anatomical Distribution of Deeply Infiltrating Endometriosis: Surgical Implications and Proposition for a Classification. Hum. Reprod..

[3] Leone Roberti Maggiore U., Ferrero S., Candiani M., Somigliana E., Viganò P., Vercellini P. (2017). Bladder Endometriosis: A Systematic Review of Pathogenesis, Diagnosis, Treatment, Impact on Fertility, and Risk of Malignant Transformation. Eur. Urol..

[4] Abrao M.S., Dias J.A., Bellelis P., Podgaec S., Bautzer C.R., Gromatsky C. (2009). Endometriosis of the Ureter and Bladder Are Not Associated Diseases. Fertil. Steril..

[5] Hudelist G., Ballard K., English J., Wright J., Banerjee S., Mastoroudes H., Thomas A., Singer C.F., Keckstein J. (2011). Transvaginal Sonography vs. Clinical Examination in the Preoperative Diagnosis of Deep Infiltrating Endometriosis. Ultrasound Obstet. Gynecol..

[6] Maccagnano C., Pellucchi F., Rocchini L., Ghezzi M., Scattoni V., Montorsi F., Rigatti P., Colombo R. (2012). Diagnosis and Treatment of Bladder Endometriosis: State of the Art. Urol. Int..

[7] Fedele L., Bianchi S., Zanconato G., Bergamini V., Berlanda N., Carmignani L. (2005). Long-Term Follow-up after Conservative Surgery for Bladder Endometriosis. Fertil. Steril..

[8] Pérez-Utrilla Pérez M., Aguilera Bazán A., Alonso Dorrego J.M., Hernández A., de Francisco M.G., Martín Hernández M., de Santiago J., de la Peña Barthel J. (2009). Urinary Tract Endometriosis: Clinical, Diagnostic, and Therapeutic Aspects. Urology.

[9] Antonelli A., Simeone C., Zani D., Sacconi T., Minini G., Canossi E., Cunico S.C. (2006). Clinical Aspects and Surgical Treatment of Urinary Tract Endometriosis: Our Experience with 31 Cases. Eur. Urol..

[10] Terho A.M., Mäkelä-Kaikkonen J., Ohtonen P., Uimari O., Puhto T., Rautio T., Koivurova S. (2022). Robotic versus Laparoscopic Surgery for Severe Deep Endometriosis: Protocol for a Randomised Controlled Trial (ROBEndo Trial). BMJ Open.

[11] Cela V., Malacarne E., Braganti F., Papini F. (2020). Robotic Surgery for Endometriosis. Gynecol. Pelvic Med..

[12] Mikhail E., Pavlovic Z.J., Al Jumaily M., Kheil M.H., Moawad G.N., Soares T. (2022). Robot-Assisted Surgery for Endometriosis Current and Future Perspectives. Surg. Technol. Int..

[13] Keckstein J., Becker C.M., Canis M., Feki A., Grimbizis G.F., Hummelshoj L., Nisolle M., Roman H., Saridogan E., Working group of ESGE, ESHRE, and WES (2020). Recommendations for the Surgical Treatment of Endometriosis. Part 2: Deep Endometriosis. Hum. Reprod. Open.

[14] Selçuk İ., Ersak B., Tatar İ., Güngör T., Huri E. (2018). Basic Clinical Retroperitoneal Anatomy for Pelvic Surgeons. Turk. J. Obstet. Gynecol..

[15] Devi Y., Singh N., Singh C., Devi L., Guria L. (2019). Morphogenesis of Urinary Bladder in Human Fetuses. J. Med. Soc. N. J..

[16] Shinagare A.B., Sadow C.A., Sahni V.A., Silverman S.G. (2011). Urinary Bladder: Normal Appearance and Mimics of Malignancy at CT Urography. Cancer Imaging.

[17] Cattaneo F., Motterle G., Zattoni F., Morlacco A., Dal Moro F. (2018). The Role of Lymph Node Dissection in the Treatment of Bladder Cancer. Front. Surg..

[18] Roy H.A., Green A.L. (2019). The Central Autonomic Network and Regulation of Bladder Function. Front. Neurosci..

[19] Page M.J., McKenzie J.E., Bossuyt P.M., Boutron I., Hoffmann T.C., Mulrow C.D., Shamseer L., Tetzlaff J.M., Akl E.A., Brennan S.E. (2021). The PRISMA 2020 Statement: An Updated Guideline for Reporting Systematic Reviews. BMJ.

[20] Koninckx P.R., Fernandes R., Ussia A., Schindler L., Wattiez A., Al-Suwaidi S., Amro B., Al-Maamari B., Hakim Z., Tahlak M. (2021). Pathogenesis Based Diagnosis and Treatment of Endometriosis. Front. Endocrinol..

[21] Saget E., Peschot C., Bonin L., Belghiti J., Boulland E., Ghesquiere L., Golfier F., Hebert T., Kerbage Y., Lavoue V. (2022). Robot-Assisted Laparoscopy for Deep Infiltrating Endometriosis: A Retrospective French Multicentric Study (2008–2019) Using the Society of European Robotic Gynecological Surgery Endometriosis Database. Arch. Gynecol. Obstet..

[22] Soto E., Luu T.H., Liu X., Magrina J.F., Wasson M.N., Einarsson J.I., Cohen S.L., Falcone T. (2017). Laparoscopy vs. Robotic Surgery for Endometriosis (LAROSE): A Multicenter, Randomized, Controlled Trial. Fertil. Steril..

[23] Nezhat F.R., Sirota I. (2014). Perioperative Outcomes of Robotic Assisted Laparoscopic Surgery versus Conventional Laparoscopy Surgery for Advanced-Stage Endometriosis. JSLS.

[24] Guan Z., Soni S.D., Zhou J., Sunkara S., Guan X. (2022). Cystoscopic-Guided Robotic Resection of Bladder Trigone Endometriosis Nodule with Ureteral Preservation. J. Minim. Invasive Gynecol..

[25] Bahadur A., Mundhra R., Sherwani P., Kumar S. (2021). Robot-Assisted Partial Cystectomy for Bladder Endometriosis: Dual Approach Involving Cystoscopy and Robotic Surgery. BMJ Case Rep..

[26] Tamhane N., Wiegand L., Mikhail E. (2020). Robotic Excision of Deep Infiltrating Endometriosis at the Uretero-Vesical Junction. Int. Braz. J. Urol..

[27] Abo C., Roman H., Bridoux V., Huet E., Tuech J.-J., Resch B., Stochino E., Marpeau L., Darwish B. (2017). Management of Deep Infiltrating Endometriosis by Laparoscopic Route with Robotic Assistance: 3-Year Experience. J. Gynecol. Obstet. Hum. Reprod..

[28] Wei Y., Su J., Zhu Q., Guan Q. (2021). Application of Transvesical Robotic Laparoendoscopic Single-Site Surgery for an Endometrial Cyst over the Urinary Bladder. Asian J. Surg..

[29] Chammas M.F., Kim F.J., Barbarino A., Hubert N., Feuillu B., Coissard A., Hubert J. (2008). Asymptomatic Rectal and Bladder Endometriosis: A Case for Robotic-Assisted Surgery. Can. J. Urol..

[30] Liu C., Perisic D., Samadi D., Nezhat F. (2008). Robotic-Assisted Laparoscopic Partial Bladder Resection for the Treatment of Infiltrating Endometriosis. J. Minim. Invasive Gynecol..

[31] Bot-Robin V., Rubod C., Zini L., Collinet P. (2011). Early evaluation of the feasibility of robot-assisted laparoscopy in the surgical treatment of deep infiltrating endometriosis. Gynecol. Obstet. Fertil..

[32] Moawad G., Tyan P., Marfori C., Abi Khalil E., Park D. (2019). Effect of Postoperative Partial Bladder Filling after Minimally Invasive Hysterectomy on Postanesthesia Care Unit Discharge and Cost: A Single-Blinded, Randomized Controlled Trial. Am. J. Obstet. Gynecol..

[33] Tan S.-J., Chen C.-H., Yeh S.-D., Lin Y.-H., Tzeng C.-R. (2018). Pregnancy Following Robot-Assisted Laparoscopic Partial Cystectomy and Gonadotropin-Releasing Hormone Agonist Treatment within Three Months in an Infertile Woman with Bladder Endometriosis. Taiwan. J. Obstet. Gynecol..

[34] Sener A., Chew B.H., Duvdevani M., Brock G.B., Vilos G.A., Pautler S.E. (2006). Combined Transurethral and Laparoscopic Partial Cystectomy and Robot-Assisted Bladder Repair for the Treatment of Bladder Endometrioma. J. Minim. Invasive Gynecol..

[35] Moawad G.N., Tyan P., Abi Khalil E.D., Samuel D., Obias V. (2018). Multidisciplinary Resection of Deeply Infiltrative Endometriosis. J. Minim. Invasive Gynecol..

[36] Siesto G., Ieda N., Rosati R., Vitobello D. (2014). Robotic Surgery for Deep Endometriosis: A Paradigm Shift. Int. J. Med. Robot..

[37] Di Maida F., Mari A., Morselli S., Campi R., Sforza S., Cocci A., Tellini R., Tuccio A., Petraglia F., Masieri L. (2020). Robotic Treatment for Urinary Tract Endometriosis: Preliminary Results and Surgical Details in a High-Volume Single-Institutional Cohort Study. Surg. Endosc..

[38] le Carpentier M., Merlot B., Bot Robin V., Rubod C., Collinet P. (2016). Partial cystectomy for bladder endometriosis: Robotic assisted laparoscopy versus standard laparoscopy. Gynecol. Obstet. Fertil..

[39] Philip C.-A., Froc E., Chapron C., Hebert T., Douvier S., Filipuzzi L., Descamps P., Agostini A., Collinet P., von Theobald P. (2021). Surgical Management of Urinary Tract Endometriosis: A 1-Year Longitudinal Multicenter Pilot Study at 31 French Hospitals (by the FRIENDS Group). J. Minim. Invasive Gynecol..

[40] Nezhat C., Hajhosseini B., King L.P. (2011). Robotic-Assisted Laparoscopic Treatment of Bowel, Bladder, and Ureteral Endometriosis. JSLS.

[41] Crispi C.P., de Souza C.A.P., Oliveira M.A.P., Dibi R.P., Cardeman L., Sato H., Schor E. (2012). Endometriosis of the Round Ligament of the Uterus. J. Minim. Invasive Gynecol..

[42] Oliveira M.A.P., Crispi C.P., Oliveira F.M., Junior P.S., Raymundo T.S., Pereira T.D. (2014). Double Circular Stapler Technique for Bowel Resection in Rectosigmoid Endometriosis. J. Minim. Invasive Gynecol..

[43] Ercoli A., Delmas V., Fanfani F., Gadonneix P., Ceccaroni M., Fagotti A., Mancuso S., Scambia G. (2005). Terminologia Anatomica versus unofficial descriptions and nomenclature of the fasciae and ligaments of the female pelvis: A dissection-based comparative study. Am. J. Obstet. Gynecol..

